# Rapid pollutant degradation by peroxymonosulfate *via* an unusual mediated-electron transfer pathway under spatial-confinement†

**DOI:** 10.1039/d1ra08954d

**Published:** 2022-02-11

**Authors:** Siting Shao, Jiahao Cui, Lina Li, Mingqi Wang, Peng Zhang, Jianguo Cui, Chun Hu, Yubao Zhao

**Affiliations:** Key Laboratory for Water Quality and Conservation of the Pearl River Delta, Ministry of Education, Institute of Environmental Research at Greater Bay Area, Guangzhou University 510006 Guangzhou P. R. China ybzhao@gzhu.edu.cn; Baotou Research Institute of Rare Earths 014030 Baotou P. R. China

## Abstract

Nano-confinement systems offer various extraordinary chemical/physical properties, due to the spatial restriction and the electronic interaction between the confined species and the surrounding medium. They are, therefore, providing rich opportunities for the design of efficient catalytic reaction systems for pollutant removal. Herein, a highly efficient mediated-electron transfer pathway is identified on a spatially-confined zero valent cobalt for abatement of the organic pollutants by PMS. The catalyst showed efficient catalytic performance in both batch and a flow reactor for degradation of various pollutants, *e.g.*, a degradation reaction constant of 0.052 s^−1^ for sulfamethoxazole and 0.041 s^−1^ for BPA. Regulated by the spatial-confinement, a distinctive inverse relationship between PMS decomposition rate and the electron density of the pollutant molecule was experimentally substantiated, *e.g.*, in the presence of the electron-rich sulfamethoxazole, PMS decomposed slower than that with BPA, while in the presence of electron deficient diphenhydramine, PMS decomposed faster than that with BPA. The unique reaction mechanism endows the spatially-confined cobalt with the capability of eliminating the priority pollutants in the complex water matrix with pervasive halide ions and natural organic matter (NOM) *via* PMS activation.

## Abstract 2

A highly efficient mediated-electron transfer process of PMS activation on Co was achieved by construction of a spatially-confined reaction environment.

## Graphical abstract

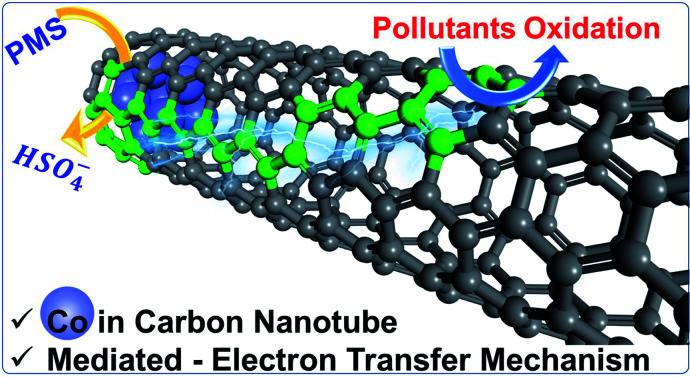

## 1. Introduction

Peroxymonosulfate (PMS) based advanced oxidation processes (AOPs) have drawn great attention as potential technology for abatement of the refractory organic pollutants in water and soil.^1–6^ Among various methods for the activation of PMS, cobalt catalysis was found to be an efficient approach, in which the reactive sulfate radicals (SO_4_˙^−^) and hydroxyl radicals (˙OH) were reckoned as the predominant reactive species.^7–9^ With the aim of efficient water remediation, various cobalt based catalysts were designed; and, notably, the nano-structure/coordination configuration of cobalt species were found to be sensitive to the catalytic performance for PMS activation. The understanding on the insights of PMS activation mechanism by cobalt species consequently underwent constant evolution. On the cobalt nanoparticles, PMS was reductively activated, generating SO_4_˙^−^ and ˙OH species which are predominant reactive species for pollutants degradation.^10^ While in the cases of single-atom cobalt catalysts with Co–N_*x*_ configurations, singlet oxygen (^1^O_2_) was identified to be the predominant reactive species from PMS activation.^11,12^ Nevertheless, SO_4_˙^−^ and ˙OH were identified to contribute predominantly to the pollutant degradation in the reaction system wherein PMS was activated by pyridine-coordinated Co single atoms embedded in a polyaromatic macrostructure.^13^ In Co(ii) catalyzed PMS activation process, high-valent cobalt-oxo species, Co(iv)=O, was proposed to be active for methyl phenyl sulfoxide oxidation.^14^ Concurrently, Co(ii)-PMS complex (Co^2+^–OOSO_3_^−^) was proposed to be the predominant reactive species according to the analysis on its capability of conducting both one-electron-transfer and oxygen-atom-transfer reactions.^15^

PMS activation mechanism varies, and the performance of the reactive species is sensitively impacted by the complexity in the water matrix as well.^16–24^ For instance, the radical pathways involve highly oxidative SO_4_˙^−^ and ˙OH (*E*^0^ (SO_4_˙^−^/SO_4_^2−^) = 2.6–3.1 V *vs.* NHE, *E*^0^ (˙OH/OH^−^) = 1.9–2.7 V *vs.* NHE), and the reaction systems are consequently vulnerable to the natural organic matters (NOMs), and the halide ions;^17,25–27^ the singlet oxygen has relatively low oxidation power (*E*^0^ (^1^O_2_/O_2_˙^−^) = 0.81 V *vs.* NHE), therefore presenting substrate-specific reactivity, such as the minimal interference from the NOMs, and selectivity towards the biomolecules.^28–31^ Therefore, it is highly desirable to control the PMS activation pathways, so as to meet the specific requirements in a real water treatment scenario. It is, however, challenging, due to the fact that the current understanding on the PMS activation mechanism is still insufficient and controversial.^16,25^ Further understanding on the structural dependence of the reaction mechanism is highly desirable.

Due to the spatial restriction and the electronic interaction between the confined species with the surrounding medium. Spatial-confinement system offers various extraordinary chemical/physical properties.^32,33^ Pan *et al.* experimentally substantiated that the Fenton reaction, an archetypal radical reaction, proceeded *via* a novel non-radical pathway with singlet oxygen as the major reactive species under nanoconfinement.^34^ The strategy of nanoconfinement is offering rich opportunities to the design of efficient catalytic reaction system for pollutants removal.^35–37^ By various nanostructure engineering strategies, the radical and singlet oxygen involved reaction pathways are hitherto realized in the cobalt catalyzed PMS activation reaction systems.^10–12^ The mediated-electron transfer reaction pathway, which usually governs the carbon and noble metals catalyzed reaction systems, is sporadically mentioned in the cobalt based catalysis system. Herein, an efficient pollutants degradation reaction system was developed based on the strategy of spatial-confinement, and an unusual mediated-electron transfer mechanism on the spatially confined Co nanocrystal is demonstrated.

## 2. Experimental section

### 2.1 Catalysts synthesis

20 grams of 2-cyanoguanidine was dissolved in 100 mL H_2_O in a reflux condensation system. 9.88 g Co(NO_3_)_2_·6H_2_O (8.487 mmol) was added in the solution and refluxed until dissolution, followed by adding 17.6 mL formaldehyde solution (36–38 wt%). The mixture was refluxed for 20 h to obtain dicyandiamide–formaldehyde polymer with Co^2+^ coordinated in the framework. The mixture was then heated on a hot-plate for removing water. Dried powder was calcined at 900 °C in argon flow, and the temperature ramping rate was 2.5 °C min^−1^. The as obtained sample was then pulverized and refluxed in 1 M sulfuric acid at 80 °C for 12 h to remove the cobalt particles outside the carbon nanotube. Due to the changed redox properties of the metals encapsulated inside the curved surface, the spatially-confined cobalt crystals were preserved in the tube after acid treatment.^38–40^ The sample was then washed until the filtrate is at circumneutral pH. The dried sample was Co-NC for catalytic reactions. Control sample without Co (denoted by NC) was synthesized in the absence of Co(NO_3_)_2_·6H_2_O *via* otherwise the same method to that for Co-NC. Co metal powder (denoted by Co-P) was purchased from Mecklin with purity of 99.9% and particle size of 300 mesh. Co_3_O_4_ was purchased from Mecklin as a bench mark catalyst.

### 2.2 Batch and flow reaction procedures

Unless otherwise specified, all the batch reactions were conducted in a 100 mL reactor; the catalyst loading was 0.15 g L^−1^, PMS concentration was 1 mM, and pollutants concentration was 20 ppm. In a typical batch reaction experiment, 7.5 mg Co-NC was dispersed in 50 mL BPA solution (20 ppm), and the reaction was started by adding 1 mL PMS (50 mM) solution. The suspension was magnetically stirred and sampled at specified time intervals; the samples were filtered and mixed with equal volume of methanol for analysis on HPLC.

### 2.3 Flow reaction conditions

7.5 mg Co-NC was mixed with silica sand and packed into a silica tube with inner diameter of 1 cm. The solution with 10 ppm BPA and 0.75 mM PMS was feed into the reactor at the flow rate of 96 mL h^−1^ by peristaltic pump. The effluent was sampled at specified interval and mixed with equal amount of methanol for analysis.

The pollutants degradation processes were analyzed by Shimadzu SIL-20A HPLC with Shim-pack GIST C18 column (4.6 × 250 mm, 5 μm). Mobile phase and detection wavelength setting for the pollutants: bis phenol A (BPA), methanol/water (70/30) and *λ* = 225 nm; sulfamethoxazole, methanol/water (55/45) and *λ* = 266 nm; 2-chloro-phenol and diphenhydramine, phosphate acid solution (0.08%)/acetonitrile (50/50) and *λ* = 221 nm; phenytoin, phosphate acid solution (0.08%)/acetonitrile (60/40) and *λ* = 220 nm; carbamazepine, methanol/water (70/30) and *λ* = 285 nm; methyl phenyl sulfoxide (MPSO), phosphate acid solution (0.08%)/acetonitrile (70/30) and *λ* = 215 nm.

## 3. Results and discussion

### 3.1 Materials synthesis and characterizations

**Fig. 1:**
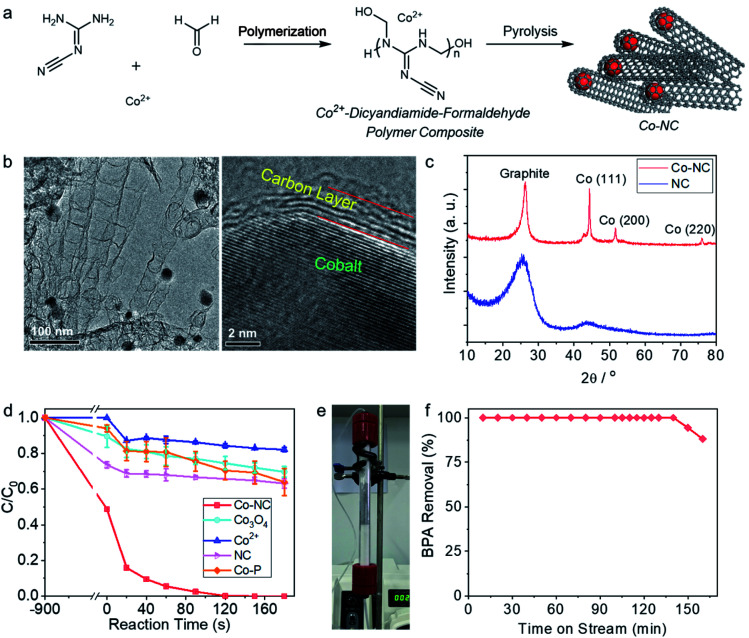
(a) Synthesis of Co-NC. (b) Transmission electron microscopy (TEM) images of Co-NC. (c) X-ray diffraction profiles of NC and Co-NC. (d) Degradation of bisphenol A (BPA) in the reaction systems with various catalyst. Reaction conditions: BPA, 20 ppm; PMS, 1 mM; initial pH, 6.5; temperature, 25 °C; catalyst loadings: Co^2+^, 0.4 mg L^−1^; Co_3_O_4_, NC, and Co-NC, 0.15 g L^−1^. (e) Photograph of the fixed bed flow reactor. (f) BPA removal performance in a fixed bed flow reactor with Co-NC as the catalyst. Reaction conditions: catalyst loading, 7.5 mg; BPA, 10 ppm; PMS, 0.75 mM; flow rate, 96 mL h^−1^.

Co-NC is synthesized by coordinative dispersion of the Co^2+^ in the polymer of melamine and formaldehyde, followed by pyrolysis of the compound (Fig. 1a). The cobalt catalysis under high temperature leads to the growth of the nitrogen-doped carbon nanotube, wherein the cobalt nanocrystals is capsulated inside the dead-end of the multilayers carbon nanotube. As shown in the TEM image (Fig. 1b), the nanotube is around 50 nm in diameter, and the encapsulated cobalt crystal with size of 20–30 nm is obviously observed by TEM. HRTEM image clearly shows the lattice of cobalt and the interface between few layers carbon and the cobalt crystal. In the X-ray diffraction (XRD) pattern (Fig. 1c), the diffraction peaks at 2*θ* of 44.2°, 51.2° and 75.8° state that the cobalt nanocrystals are in face-centered cubic (fcc) structure.^41^ As determined by nitrogen physical adsorption method, BET surface area of Co-NC is 303.2 m^2^ g^−1^, and pore sized is distributed mainly at around 2–6 nm (Fig. S1†). As shown in the X-ray photo-electron spectra (Fig. S2†), the N 1s signal is deconvoluted into three peaks at 398.3 eV, 400.6 eV, and 403.5 eV, which are attributed to pyridinic N, pyridonic N, and pyridine-*N*-oxide N, respectively.^42,43^ The C 1s signal could be deconvoluted into three peaks at 284.4 eV, 285.1 eV, and 288.5 eV, which are attributed to the carbon atoms in sp^2^ configuration, C–O group, and the adventitious carbon, respectively.^44^ O 1s peaks are deconvoluted into two peaks at 530.1 and 532.1 eV, which are assigned to surface Co–O species and the oxygen atom in organic groups.^45,46^

### 3.2 Pollutants degradation reaction performance

BPA, which is a commonly used plastic additive, but a pervasive endocrine disruptor in the aquatic systems, was employed as one of the target pollutants.^47^ Under optimized reaction conditions (Fig. S3†), 20 ppm BPA could be rapidly degraded within 120 s on Co-NC (0.15 g L^−1^) in the presence of 1 mM PMS, presenting a remarkable pseudo-first order reaction kinetic constant of 0.040 s^−1^ (Fig. 1d). The concentration of the leached Co^2+^ at 120 s was 0.26 ppm; and the catalytic performance of the leached Co^2+^ played a minor role in the overall catalytic reaction, due to the fact that 18% of the BPA was removed in the presence of 0.4 mg L^−1^ Co^2+^ in 180 s. Under the same reaction conditions, the reaction systems with Co_3_O_4_, a benchmark catalyst for PMS activation, showed only 25% BPA removal within 120 s. NC was synthesized without the addition of the cobalt ion *via* otherwise the same method to that for Co-NC, while the cobalt free NC showed poor catalytic efficiency, removing only 37% BPA in 180 s.

Humic acid is a one of the typical natural organic matters (NOMs) in the natural water matrix, and the impact of the humic acid on the catalytic performance of Co-NC was thus investigated. As shown in Fig. S4a,† the presence of humic acid slightly impeded the BPA degradation performance; in the presence of 100 mg L^−1^ humic acid, >90% of BPA could be removed within 180 s reaction. It is worth noting that the presence of humic acid in the solution decreased the BPA absorption on Co-NC, *e.g.*, 68.2 μg per mg Co-NC in the absence of humic acid and 58.0 μg per mg Co-NC in the presence of 100 ppm humic acid (Fig. S4b†); the surface adsorption of BPA on Co-NC is thus tentatively believed to be related to the degradation process. The mineral anions, which are commonly presenting in the natural water system, were added in the reaction system for checking their potential impacts on the pollutants degradation performance. BPA was efficiently eliminated in the presence of 5 mM of Cl^−^, CO_3_^2−^, H_2_PO_4_^−^, and NO_3_^−^ (Fig. S5†). Carbonate anion, in particular, showed relatively obvious negative impact on the BPA degradation reaction. Inspired by the case of humic acid influence, an investigation on the effect of CO_3_^2−^ in the BPA adsorption was conducted. Similarly, the presence of CO_3_^2−^ in the solution reduced the surface adsorption of BPA, *e.g.*, the amount of BPA adsorbed on Co-NC in the presence of 5 mM CO_3_^2−^ and in the absence of CO_3_^2−^ was, respectively, 36.1 and 68.2 μg per mg Co-NC. We thus surmise that it is the surface catalytic reaction mechanism wherein the BPA surface adsorption property matters, rather than the free radical mechanism, that is governing the pollutant degradation process in this case. The mechanism will be analyzed in the following radical quenching experiments.

For further exploring the durability of Co-NC in the BPA degradation reaction, the catalytic performance of Co-NC was evaluated with a fixed-bed flow reactor (Fig. 1e). As shown in Fig. 1f, at flow rate of 12.8 L h^−1^ (for 1 g Co-NC), the reactor run efficiently for 140 min with 100% removal of BPA, and the adsorption accounts for a small part in the BPA removal (Fig. S6†). For the benchmark catalyst of Co_3_O_4_, there is no stable BPA degradation performance observed; and the Co-free NC deactivated in 50 min in the flow reactor (Fig. S7†).

**Fig. 2:**
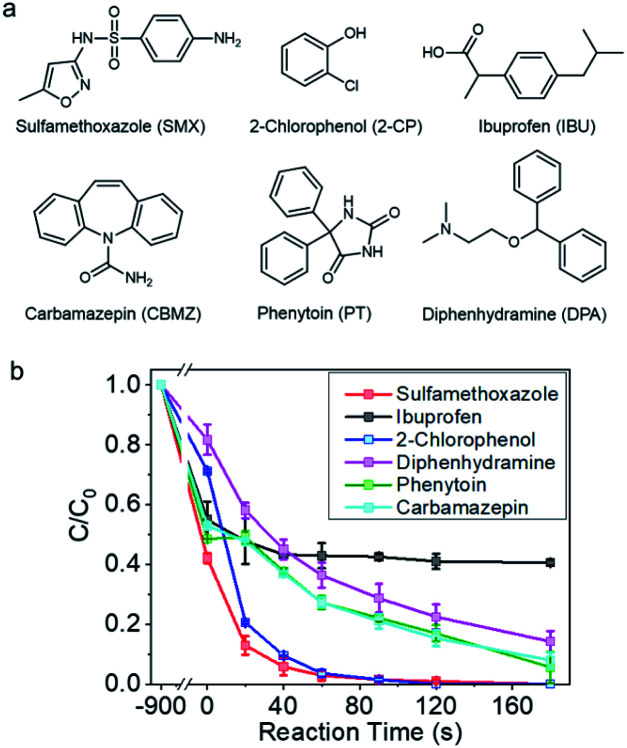
(a) The structures of selected refractory organic pollutants in water. (b) Catalytic degradation of various refractory organic pollutants by Co-NC. Reaction conditions: pollutants concentration, 20 ppm; PMS concentration, 1 mM; catalyst loading, 0.15 g L^−1^; initial pH, 6.5.

The catalyst was then employed for degrading a couple of selected refractory organic pollutants concerned by the public, such as sulfamethoxazole, carbamazepine, phenytoin, ibuprophen, and diphenhydramine, which are pharmaceuticals, and 2-chloro-phenol, a representative organohalide pollutant (Fig. 2a).^47^ As shown in Fig. 2b and S8,† sulfamethoxazole and 2-chloro-phenol can be completely removed within 120 s. Diphenhydramine, phenytoin, and carbamazepine were also vulnerable to be rapidly degraded, and more than 80% removal could be achieved in 180 s. However, ibuprofen, with electron deficient molecular structure, was unable to be degraded in this reaction system. The selectivity towards the pollutants was indicative of a non-radical reaction mechanism.

### 3.3. Mechanistic insights

**Fig. 3:**
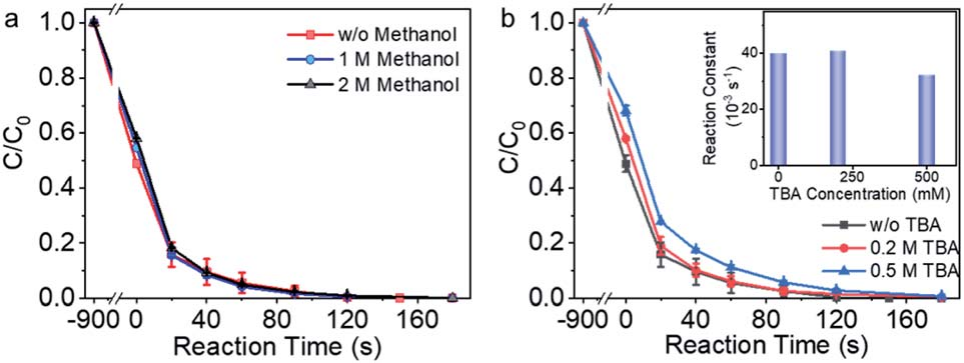
Analyzing the possibility of the radical reaction mechanism. (a) The impact of methanol on the BPA degradation performance. (b) BPA degradation performance in the presence and absence of *tert*-butyl alcohol (TBA).

Methanol and *tert*-butyl alcohol (TBA) are capable of trapping the free radicals due to the rapid reaction rate, *e.g.*, *k* (˙OH/CH_3_OH) = (1.2–2.8) ×10^9^ M^−1^ s^−1^, *k* (SO_4_˙^−^/CH_3_OH) = (1.6–7.8) ×10^7^ M^−1^ s^−1^, and *k* (˙OH/TBA) = (3.8–7.6) × 10^8^ M^−1^ s^−1^.^48,49^ Methanol and TBA were thus employed to examine the contribution of the free radicals to the pollutant degradation process in Co-NC catalyzed reaction system. As shown in Fig. 3a, in the presence of 1 M and 2 M methanol (>1 × 10^5^ times of BPA molar concentration), BPA degradation reaction rate is the same to that without methanol. In the presence of 0.2 M TBA, there was no obvious attenuation of the BPA degradation reaction rate, 20 ppm BPA was eliminated efficiently within 180 s; the BPA degradation reaction rate decreased slightly in the presence of 0.5 M TBA (>5 × 10^4^ times of the BPA concentration). The radical quenching experiments demonstrated that the radical pathway played negligible role during BPA degradation.

Electron spin resonance (ESR) was employed to detect the free radicals, such as SO_4_˙^−^ and ˙OH, with 5,5-dimethyl-1-pyrroline *N*-oxide (DMPO) as the spin-trapping agent.^50^ However, the concentration of the free radicals might be below the detection limit (Fig. S9†). The ESR trapping experiments, therefore, partially supported the aforementioned hypothetical non-radical reaction mechanism involving a critical step of BPA surface adsorption.

**Fig. 4:**
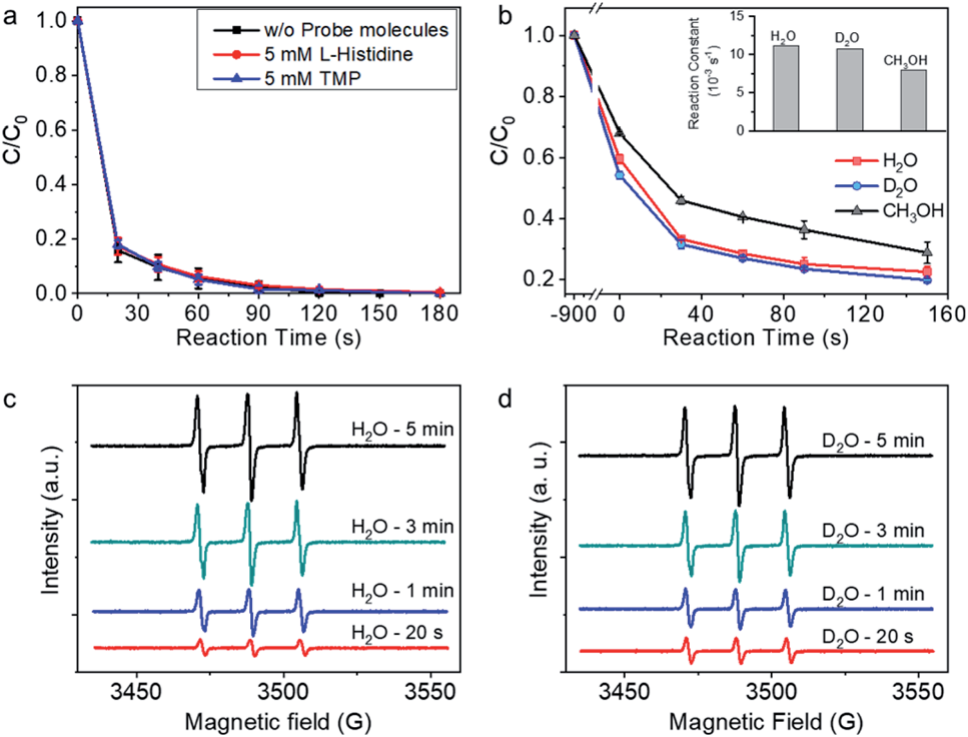
Analyzing the possible role of singlet oxygen (^1^O_2_) in the reaction system. (a) Impact of the l-histidine and 2,2,6,6-tetramethylpiperidine (TMP) on the BPA degradation performance. Reaction conditions: Co-NC, 0.15 g L^−1^; PMS, 1 mM; BPA, 20 ppm; probe molecules, 5 mM; solvent, H_2_O; initial pH, 6.5. (b) Solvent impact on the BPA degradation performance. Reaction conditions: Co-NC, 0.15 g L^−1^; PMS, 1 mM; BPA, 50 ppm; H_2_O, D_2_O, and CH_3_OH was respectively employed as solvent in each reaction; initial pH of the aqueous solution, 6.5. (c and d) Electron spin resonance spectra in H_2_O (c) and D_2_O (d) with TMP as the spin-trapping agent for ^1^O_2_. The scale of the intensity in (c) and (d) are the same.

To further explore the possible contribution of singlet oxygen (^1^O_2_) to BPA degradation, l-histidine and 2,2,6,6-tetramethylpiperidine (TMP), which are trapping agent for ^1^O_2_, were added in the reaction system during BPA degradation.^16,34,51^ As shown in Fig. 4a, the addition of 5 mM (>50 times of BPA concentration) l-histidine and TMP showed no impact on the BPA degradation rate, and BPA was completely removed in 120 s, tentatively indicating that ^1^O_2_ may not contribute to BPA degradation process. For further confirm the role of ^1^O_2_ in the reaction system, solvent impact was investigated. The life-time of ^1^O_2_ is sensitive to the property of surrounding chemical environment, *e.g.*, *k*_d_ (H_2_O) (the decomposition reaction rate constants in H_2_O) is 2.5 × 10^5^ s^−1^, *k*_d_ (D_2_O) is 1.5 × 10^5^ s^−1^, and *k*_d_ (CH_3_OH) is 1.1 × 10^5^ s^−1^.^52^ The BPA degradation performance, on condition that ^1^O_2_ was the predominant reactive species, should be higher in D_2_O and CH_3_OH than in H_2_O. However, the BPA degradation rate in D_2_O was close to that in H_2_O (Fig. 4b). With CH_3_OH as the solvent, BPA was degraded rapidly, although the reaction rate was slower than that in H_2_O and D_2_O. The experimental investigations on account of solvent-dependent ^1^O_2_ life time confirmed that ^1^O_2_ did not contribute to the BPA degradation reaction. Instead, the solvent-dependent BPA adsorption behavior on Co-NC was found to be positively related with BPA degradation; and the amount of surface adsorbed BPA per mg Co-NC in H_2_O, D_2_O, and CH_3_OH are 0.135, 0.153, and 0.107 mg, respectively. The above analysis is, repeatedly, indicative of a reaction mechanism with important BPA surface oxidation process.

For further checking the possibility of ^1^O_2_ generation during PMS activation, ESR with 2,2,6,6-tetramethylpiperidine (TMP) as the spin-trapping agent for ^1^O_2_ was performed. As shown in Fig. 4c, the characteristic triplet resonance peaks (hyperfine split constant, 16.9 G; *g* factor, 2.0054), which are corresponding to 2,2,6,6-tetramethylpiperidine-*N*-oxide (TMPO), increased with reaction time. The appearance of TMPO signal was tentatively indicating the possible trapping of ^1^O_2_ by TMP.^53,54^*In lieu* of H_2_O, D_2_O was used as the solvent for measurement, and intensified signals were expected as long as ^1^O_2_ was generated during PMS activation and trapped by TMP. However, the intensity of the spectra was close to that in H_2_O at the given reaction time, demonstrating that TMPO was produced *via* an alternative reaction mechanism, rather than ^1^O_2_ trapping by TMP.^55^ There is alternative pathway for TMP to TMPO conversion, which is initiated by one electron oxidation of TMP to TMP˙^+^. The active TMP˙^+^ radical cation then goes through the following deprotonation reaction as well as oxidation by dissolved oxygen, and finally converts to TMPO.^55,56^ In light of the experimental fact that no free radicals generated from PMS activation, it is plausible to proposed that the one-electron transfer process takes place on the surface adsorption site for TMP on Co-NC. The one-electron transfer step initiated TMPO generation on the surface is also indicative of the mediated-electron transfer mechanism.^55^

*In lieu* of PMS, peroxydisulfate (PDS) was employed for catalytic activation to degrade BPA. PDS has a more negative reduction potential (*E*_o_ (S_2_O_8_^2−^/SO_4_^2−^) = 2.01 V *vs.* NHE) than that of PMS, and the transition metals, such as Co, are selective for PMS activation.^10,57^ PDS activation are usually observed *via* a mediated electron transfer mechanism catalyzed by carbon materials and noble metals.^58–61^ With a spatial-confinement configuration, Co-NC exhibited a remarkable catalytic capability for BPA degradation *via* PDS activation, and 20 ppm BPA was eliminated in 2 min. As expected, ionic Co^2+^ showed negligible catalytic activity for PDS activation to degrade BPA, which is consistent with various reports (Fig. S10†).^10,57^ The capability of Co-NC in activation of both PMS and PDS, repeatedly, indicated the mediated-electron transfer mechanism.^10,16,57^

**Fig. 5:**
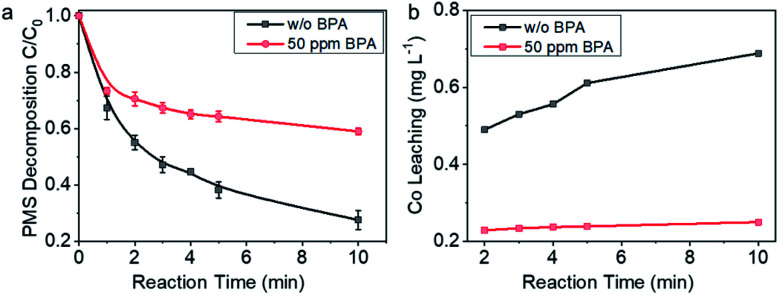
Confirmation on the mechanism of mediated electron transfer from BPA to PMS. (a) PMS decomposition behavior on Co-NC in the presence and absence of BPA. (b) Co^2+^ leaching with reaction time in the presence and absence of BPA. Reaction conditions: PMS concentration, 1 mM; 50 mL BPA solution with concentration of 50 ppm; Co-NC concentration, 0.15 g L^−1^.

For further understanding the insights of electron transfer on the interfaces in PMS–Co-NC–BPA system, PMS decomposition behavior on Co-NC was investigated. As shown in Fig. 5a, PMS decomposed rapidly on Co-NC in the absence of BPA, and >70% of PMS decomposed within 10 min. While in the presence of 50 ppm BPA in the reaction system, the PMS decomposition reaction was significantly attenuated, and 41% PMS was depleted within 10 min. The impacts of the pollutants on the PMS decomposition behavior on Co-NC were further investigated in the presence of sulfamethoxazole and diphenhydramine, which, respectively, showed faster and slower degradation kinetics than BPA. It is worth noting that in the presence of the electron-rich sulfamethoxazole, PMS decomposed slower than that with BPA; while in the presence of diphenhydramine, PMS decomposed faster than that with BPA (Fig. S11†). The case here is contrary to the known mediated electron transfer process in carbon and noble metal catalyzed reactions wherein the addition of the pollutant accelerates PMS decomposition.^10,58,60–62^ The sharp contrast herein indicates that the spatial-confinement of Co nanocrystals may alters the electron transfer process from pollutants to PMS.

Co^2+^ leaching behavior was monitored for further understanding the electron transfer process. As shown in Fig. 5b, in the absence of BPA, Co^2+^ concentration increased with reaction time, and the concentration reached 0.69 ppm with 10 min reaction. In contrast, Co^2+^ concentration in the reaction system with 50 ppm BPA was almost stable, *e.g.*, 0.23 ppm at 2 min and 0.25 ppm at 10 min. These data are indicating that the spike of BPA changed the electron transfer pathway: in the absence of BPA, there was direct interaction between Co^0^ and PMS, and electron transfer process started upon contacting, leading to the diffusion of Co^2+^ into the solvent. While in the case with 50 ppm BPA, Co nanocrystal was no longer the electron donor, but played a distinct role of conductor, transferring the electrons coming from BPA to PMS. We thus tentatively propose that BPA was oxidized by PMS with Co-NC as an electron transfer platform, which favorably improved the efficiency of PMS. For such an efficient electron transfer process, detailed insights into the relationship between microstructure and the unique electron transfer behavior is highly desired and expected to inspire the design of high-performance catalysts. It drives us to further explore the details about the catalytic process on Co-NC.

**Fig. 6:**
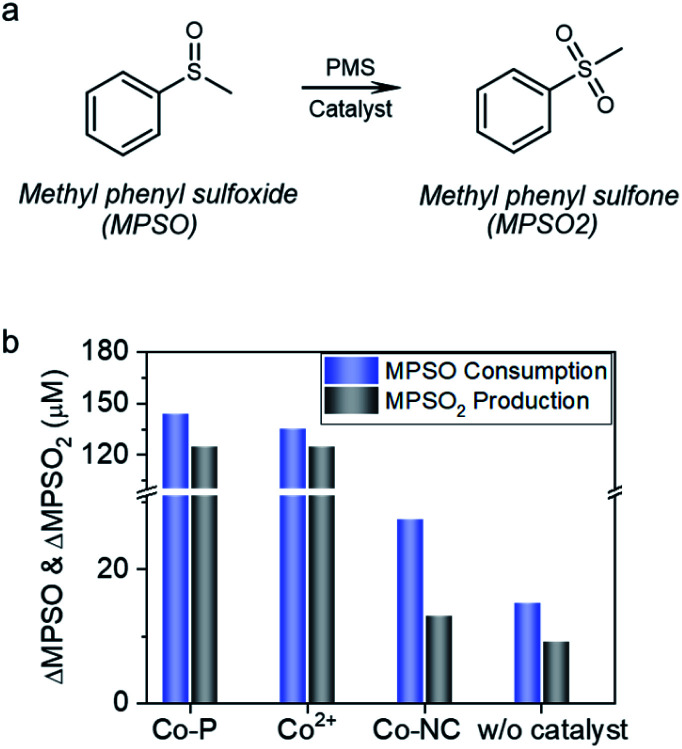
(a) Reaction equation of MPSO to MPSO_2_ probe reaction. (b) MPSO to MPSO_2_ conversion under different reaction conditions. Reaction conditions, 50 mL solution with 1 mM PMS and 0.2 mM MPSO; Co-P, 8.0 mg L^−1^; Co^2+^, 3.8 mg L^−1^; Co-NC, 150 mg L^−1^ (Co, 15.6 mg L^−1^); circumneutral pH; 5 min reaction at 25 °C.

In the Co^2+^ catalyzed reaction systems, the interaction between cobalt and PMS generated cobalt active species; *via* oxygen transfer reaction with the cobalt active species, methyl phenyl sulfoxide (MPSO) was converted to methyl phenyl sulfone (MPSO_2_) (Fig. 6a).^14,15^ The observation of MPSO to MPSO_2_ conversion demonstrated the accessibility of reactive species by MPSO.^19,63^ As shown in Fig. 6b, Co-P showed high catalytic activity for MPSO to MPSO_2_ conversion, and 5 min reaction afforded both high conversion of 71.9% and remarkable selectivity of 86.9%. During MPSO oxidation, cobalt leached gradually into the solution, and the concentration of Co^2+^ reached 2.1 ppm at the end of the reaction (Fig. S12†). To figure out the role of Co^0^ for MPSO oxidation, a reaction catalyzed by 3.8 ppm Co^2+^ was carried out for a comparison; notwithstanding with a high Co^2+^ concentration (∼2 times of the final Co^2+^ concentration in Co-P catalyzed reaction system), 5 min reaction afforded a relatively lower conversion of 67.4%. Zero valent cobalt was thus proven to be capable of catalyzing MPSO oxidation by PMS to generate MPSO_2_.

In stark contrast to the catalytic performance of Co-P, Co-NC is almost inactive for MPSO to MPSO_2_ conversion. Specifically, Co-NC catalysis for 5 min offered a low conversion of 13.7%, and poor MPSO2 selectivity of 47.3%. In light of the facts that a blank reaction without catalyst presented 7.5% conversion and 61.4% MPSO_2_ selectivity, the contribution of Co-NC catalysis in MPSO to MPSO_2_ conversion is negligible. In other words, PMS direct oxidation reaction contributes predominantly to the MPSO_2_ production in Co-NC case, indicating that MPSO oxidation on Co-NC proceeded through an alternative electron transfer pathway wherein MPSO has no access to the spatially-confined cobalt specie, thus very low selectivity towards MPSO_2_.

In light of the aforementioned experimental evidences of (1) BPA adsorption being critical for degradation, (2) TMPO formation initiated by one-electron oxidation, (3) capability in both PMS and PDS activation, and (4) negligible MPSO2 production by Co-NC catalysis, it is plausible to propose that mediated-electron transfer mechanism is governing the MPSO oxidation reaction on Co-NC. However, the details about the interaction between carbon nanotube and spatially-confined cobalt crystal, and its role in tuning the electronic properties of the cobalt crystal are still unknown and deserve further investigations.

## 4. Conclusions

Cobalt catalyzed PMS activation is reckoned as a highly efficient process for refractory organic pollutants depletion. Mechanistic understanding on the insights of the nano-structure dependent PMS activation process is instructive to the design of efficient catalysts for dealing with the specific requirements from the complex water matrix. Under spatial-confinement, the reaction process in cobalt catalysis system was adjusted to the mediated electron transfer mechanism, rather than the known radicals or singlet oxygen involved reaction. This endows the reaction system with competitively favorable capability in selectively abatement of the priority pollutants in complex water matrix with NOMs and halide ions.

## Author contributions

S. Shao: methodology, formal analysis, investigation. J. Cui: methodology, formal analysis. L. Li: methodology, writing – original draft. M. Wang: methodology, formal analysis. P. Zhang: methodology, formal analysis, writing – review & editing. J. G. Cui: formal analysis. C. Hu: resources, writing – review & editing, funding acquisition. Y. Zhao: conceptualization, formal analysis, writing – review & editing, supervision, funding acquisition.

## Conflicts of interest

There are no conflicts to declare.

## Supplementary Material

RA-012-D1RA08954D-s001
