# Supplementary material for: Rapid pollutant degradation by peroxymonosulfate via an unusual mediated-electron transfer pathway under spatial-confinement
Source: RSC Adv. 2022 Feb 11;12(9):5236–44. doi: 10.1039/d1ra08954d (PMC8981504; doi:10.1039/d1ra08954d)
Supplement: RA-012-D1RA08954D-s001 [file RA-012-D1RA08954D-s001.pdf]

Electronic Supplementary Material (ESI) for RSC Advances.
This journal is © The Royal Society of Chemistry 2022

Electronic Supplementary Information (ESI)

Rapid Pollutants Degradation by Peroxymonosulfate via an Unusual Mediated-electron Transfer Pathway under Spatial-confinement

*Siting Shao[1], Jiahao Cui,[1]Lina Li[1], Mingqi Wang[1], Peng Zhang[1], Jianguo Cui[2], Chun Hu[1], and Yubao Zhao[1]**

[1] Key Laboratory for Water Quality and Conservation of the Pearl River Delta, Ministry of Education; Institute of Environmental Research at Greater Bay, Guangzhou University, 510006 Guangzhou, P. R. China

[2] Baotou Research Institute of Rare Earths, 014030 Baotou, P. R. China

Email: ybzhao@gzhu.edu.cn (Y.Z.)

Materials characterizations:

The morphology of the catalyst sample was characterized on a JEOL 2100 transmission electron microscope (TEM).

X-ray diffraction (XRD) patterns of the catalysts were obtained on a Rigaku D/MAX 2500 diffractometer with Cu radiation (Cu Kα = 0.15406 nm).

Chemical elements were identified by x-ray photoelectron spectroscope (XPS), and data were collected on an ESCA laboratory 220i-XL spectrometer; all the binding energy were calibrated to C 1s peak at 284.6 eV.

Cobalt concentration was analyzed Inductively Coupled Plasma - Optical Emission Spectrometer (ICP-OES) (PerkinElmer, AVIO 200).

Nitrogen physisorption measurement was conducted on QuadraSorb SI via nitrogen sorption at 77 K; and the samples were degassed at 200 °C before nitrogen adsorption.

Electron spin resonance (ESR) experiments:

DMPO (5,5-Dimethyl-2-pyrrolidone-N-oxyl) was employed as the spin-trapping agent for the free radicals in the bulk solution. In a typical experiment, 0.22 mL DMPO was added into 11 mL PMS solution with concentration of 1 mM in a glass vial with volume of 15 mL under magnetic stirring. One milliliter solution was then sampled and measured by ESR as the blank experiment. 1.5 mg catalyst was then added in the glass vial to start the reaction. The samples with reaction time 20 s, 1 min, 3min, and 5 min was filtered and sealed in a capillary tube for ESR data collection.

For detecting the singlet oxygen ($^1O_2$) in the reaction system, 2,2,6,6-tetramethylpiperidine (TMP) was employed as the spin-trapping agent. In a typical experiment, 11 mL solution with 1 mM PMS, 10 mM TMP, and 20 ppm BPA was magnetically stirred in a glass via with capacity of 15 mL. 7.5 mg Co-NC was added in the glass vial to start the reaction. At specified interval, 1 mL suspension was sampled, filtered, and sealed in a capillary tube for ESR data collection.

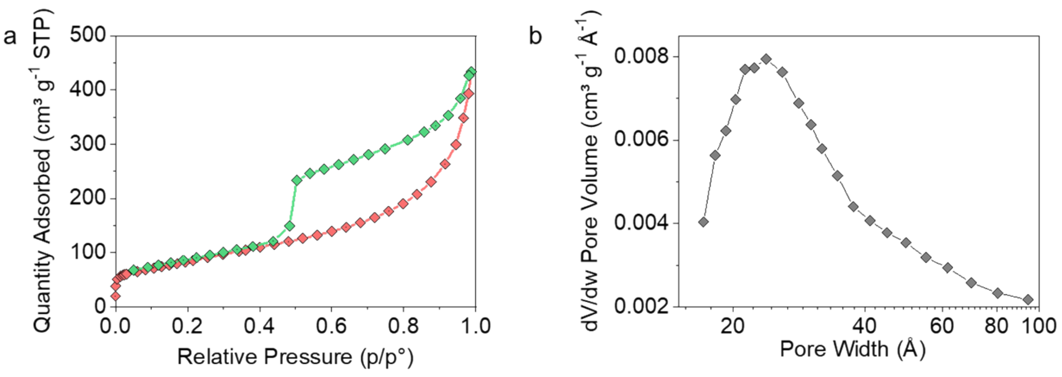

Figure S1. N_2 adsorption-desorption isotherm (a) and BJH pore size distribution profile (b) of Co-NC.

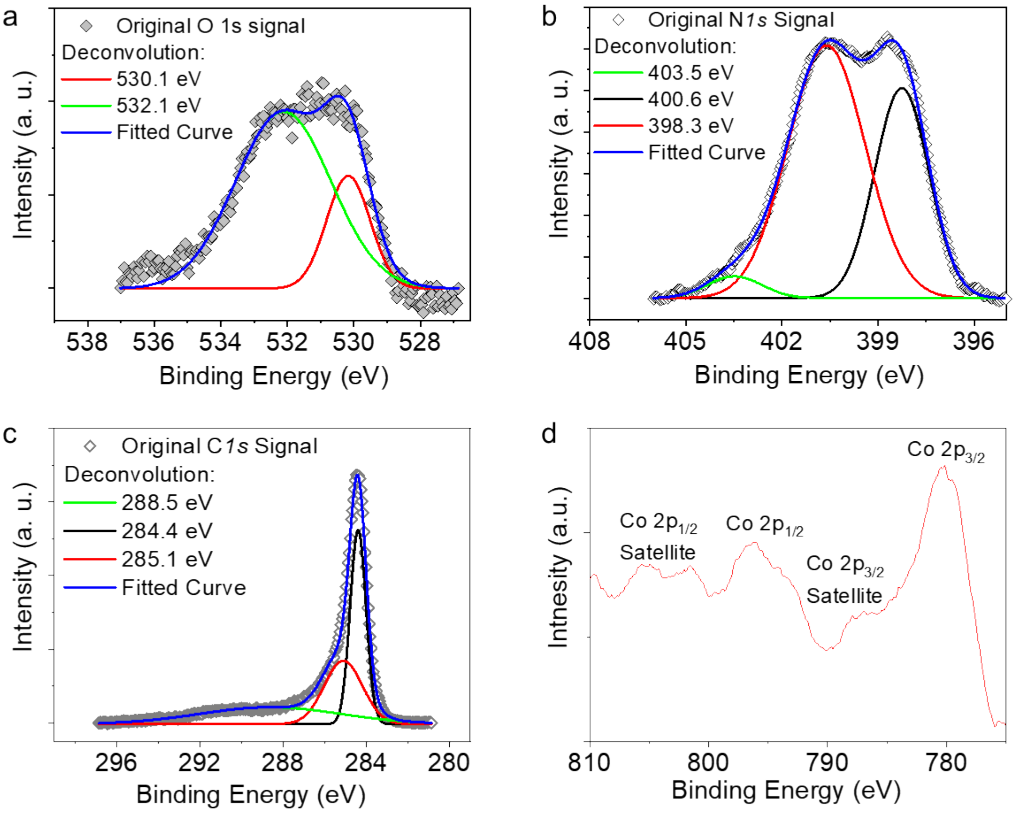

Figure S2. X-ray photo-electron spectroscopy profiles of Co-NC. (a) O 1s signal and the deconvoluted peaks. (b) N 1s signal and the deconvoluted peaks. (c) C 1s signal and the deconvoluted peaks. (d) Co 2p signal.

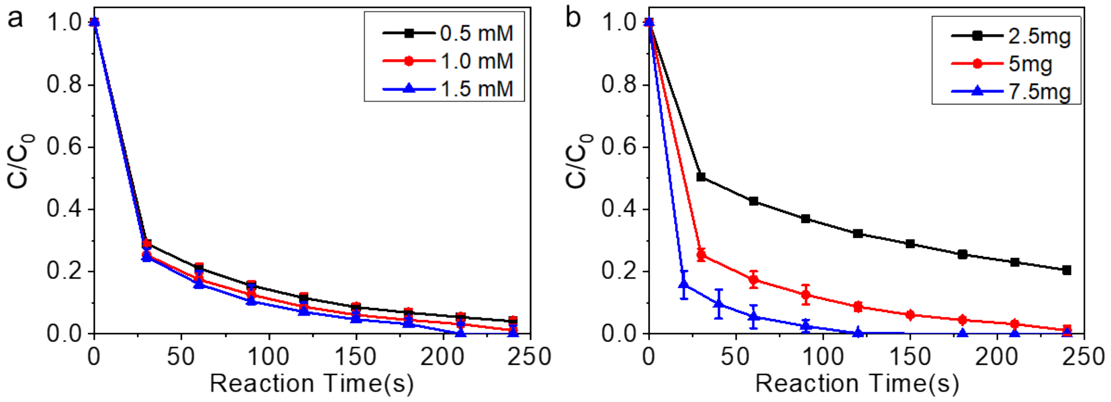

Figure S3. Reaction condition optimization. (a) BPA degradation performance at various PMS concentrations with Co-NC concentration of 0.1 g L^{-1} and BPA concentration of 20 ppm. (b) BPA degradation performance at various catalyst concentrations with PMS concentration of 1 mM and BPA concentration of 20 ppm.

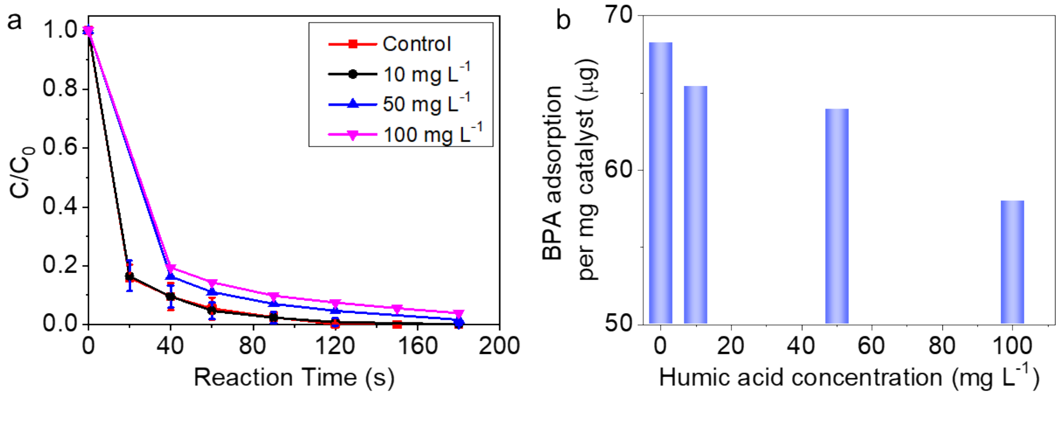

Figure S4. (a) Impact of the humic acid in the reaction suspension on BPA degradation. Reaction conditions: BPA concentration, 20 ppm; PMS concentration, 1 mM; Co-NC loadings, 7.5 mg in 50 mL aqueous suspensions; initial pH, 6.5. (b) The adsorption of BPA on Co-NC in a solution with 20 ppm BPA and various concentrations of humic acid.

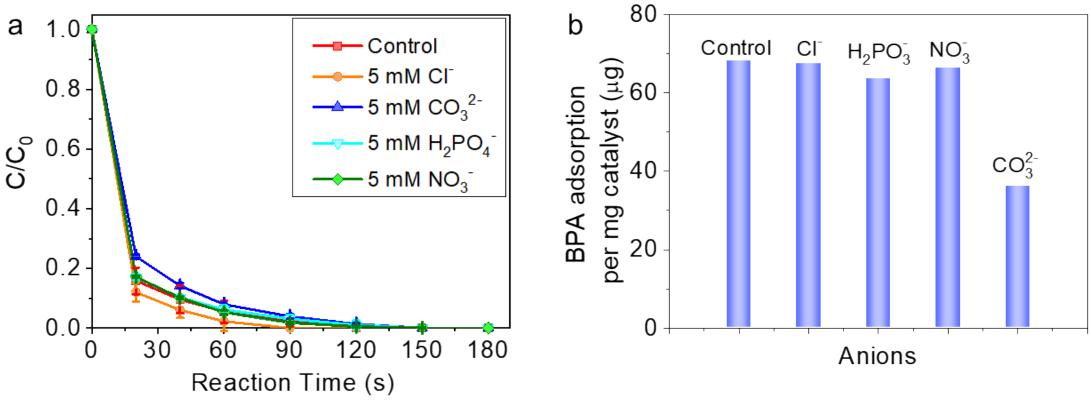

Figure S5. Impact of the anions in the reaction suspension on BPA degradation. Reaction conditions: BPA concentration, 20 ppm; PMS concentration, 1 mM; Co-NC loadings, 7.5 mg in 50 mL aqueous suspensions; initial pH, 6.5. (b) The adsorption of BPA on Co-NC in 20 ppm BPA and various anions with concentration of 5 mM.

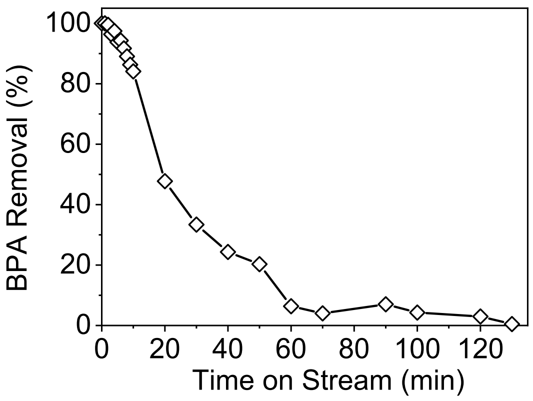

Figure S6. BPA removal by adsorption in a fixed bed reactor with Co-NC in the absence of PMS. Reaction conditions: catalyst loading, 7.5 mg; BPA, 10 ppm; flow rate, 96 mL h^{-1}.

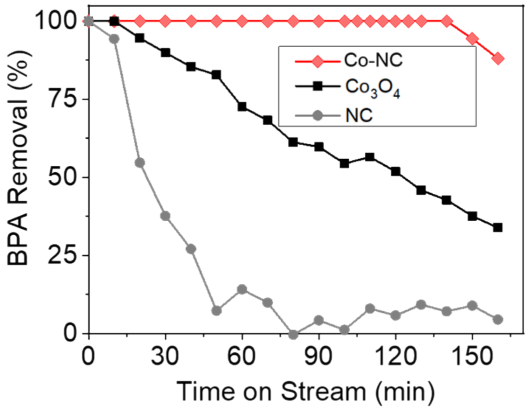

Figure S7. BPA removal performance of in a fixed bed reactor with Co-NC, Co_3O_4, and NC as the catalyst. Reaction conditions: catalyst loading, 7.5 mg; BPA, 10 ppm; PMS, 0.75 mM; flow rate, 96 mL h^{-1}.

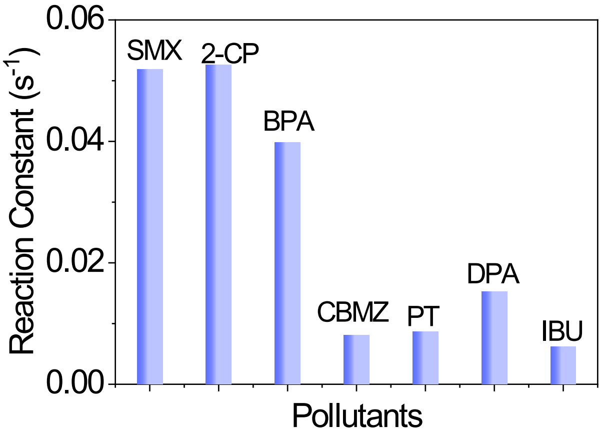

Figure S8. Pseudo-first order reaction constants of various refractory organic pollutants degradation reactions on Co-NC. Reaction conditions: pollutants concentration, 20 ppm; PMS concentration, 1 mM; initial pH, 6.5; catalyst loading, 0.16 g L^{-1}.

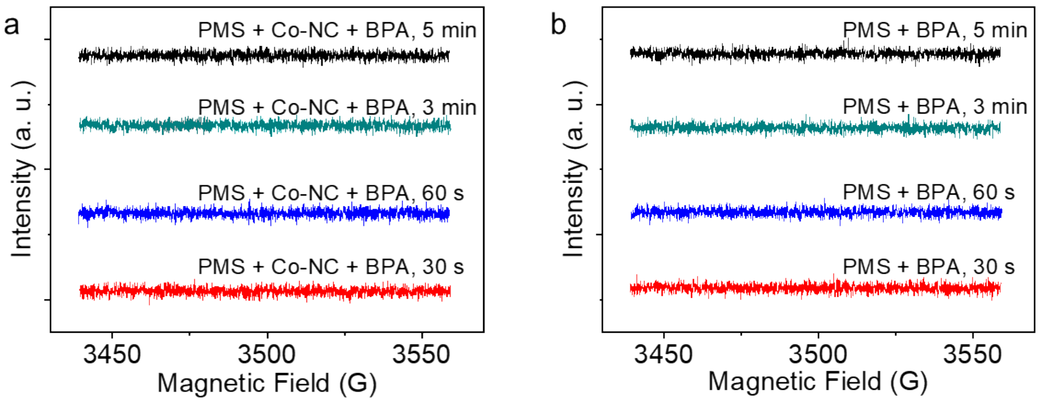

Figure S9. Electron spin resonance spectra with DMPO (5,5-Dimethyl-1-pyrroline N-oxide) as the free-radical spin-trapping agent. (a) the reaction system composed of 1 mM PMS, 0.14 mM DMPO, 0.16 g L^{-1} Co-NC, and 20 ppm BPA. (b) The reaction suspension with 1 mM PMS, 0.14 mM DMPO, and 20 ppm BPA.

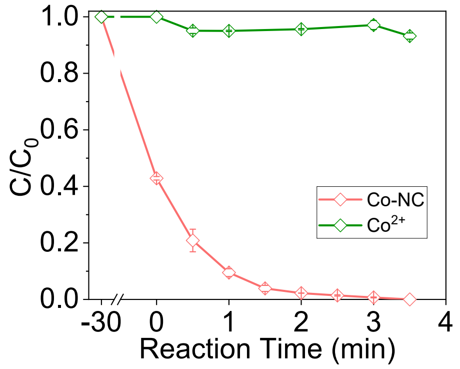

Figure S10. Catalytic degradation of BPA in the presence of PDS. Reaction conditions: BPA, 20 ppm; PDS, 1 mM; initial pH, 6.5; temperature, 25 °C; catalyst loadings: Co^{2+}, 0.26 mg L^{-1}; Co-NC, 0.15 g L^{-1}.

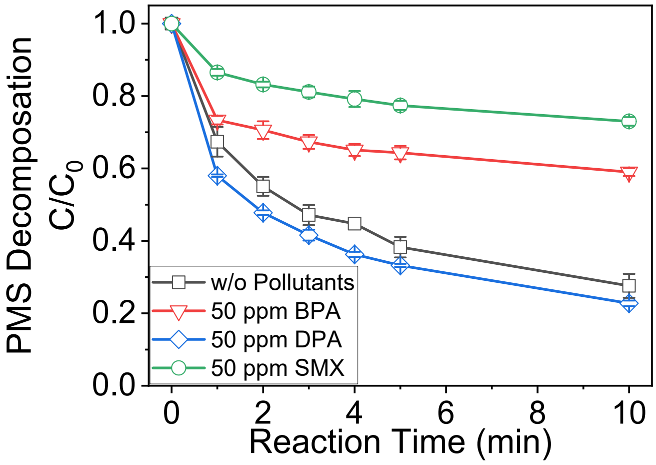

Figure S11. The impacts of the pollutants on the PMS decomposition behavior on Co-NC. Reaction conditions: PMS, 1 mM; BPA, 50 ppm; Co-NC, 0.15 g L^{-1}; initial pH, 6.5.

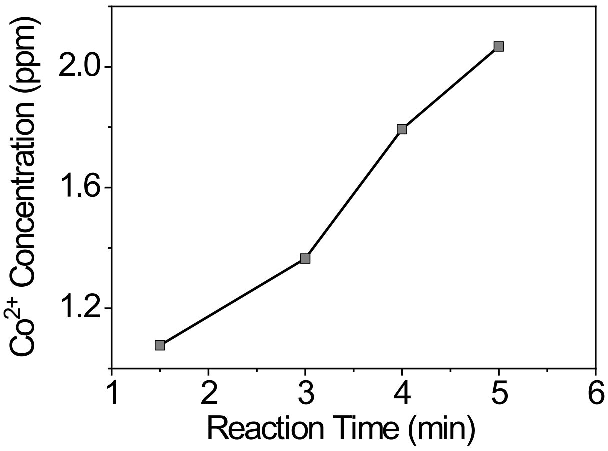

Figure S12. Co^{2+} leaching in the PMSO oxidation reaction catalyzed by Co-P in the presence of PMS. Reaction conditions: Co-P, 8 mg L^{-1}; PMS, 1 mM; PMSO, 0.2 mM; initial pH, 6.5.
